# Hereditary Endometrial Cancer: Lynch Syndrome, Mismatch Repair Deficiency, and Emerging Genetic Predispositions—A Comprehensive Review with Clinical and Laboratory Guidelines

**DOI:** 10.3390/ijms27031304

**Published:** 2026-01-28

**Authors:** Andrzej Kluk, Hanna Gryczka, Małgorzata Braszka, Rafał Ałtyn, Hanna Markiewicz, Jan K. Ślężak, Ewa Dwojak, Joanna Czerniak, Paweł Zieliński, Bartosz J. Płachno, Paula Dobosz

**Affiliations:** 1 Department of Pathomorphology, Poznan University of Medical Sciences, 60-355 Poznan, Poland; akluk@interia.pl (A.K.); jankazimierzslezak@gmail.com (J.K.Ś.); torakochirurg.zielinski@gmail.com (P.Z.);; 2 Cairns Base Hospital, Cairns, QLD 4870, Australia; 3 IT Department, Poznan University of Medical Sciences, 61-701 Poznan, Poland; 4 Department of Histology and Embryology, Faculty of Medicine, Medical University of Warsaw, 02-004 Warsaw, Poland; 5 Department of Methodology, Faculty of Medicine, Medical University of Warsaw, 02-004 Warsaw, Poland; 6 Centre of Experimental Medicine, Poznan University of Medical Sciences, 60-806 Poznan, Poland; 7 Institute of Botany, Faculty of Biology, Jagiellonian University, 30-387 Cracow, Poland; bartosz.plachno@uj.edu.pl

**Keywords:** endometrial cancer, Lynch syndrome, hereditary cancer syndromes, mismatch repair deficiency, genetic predisposition

## Abstract

Endometrial cancer is the most common gynaecologic malignancy in high-income countries, with a rising incidence largely driven by reproductive factors, obesity, and prolonged exposure to unopposed oestrogens. Although most cases are sporadic, approximately 2–5% are associated with hereditary cancer syndromes, of which Lynch syndrome represents the most important contributor. Lynch syndrome results from germline mutations in DNA mismatch repair (MMR) genes and is associated with a substantially increased lifetime risk of endometrial cancer, reaching up to 71% in carriers of MutS homologue 6 (MSH6) mutations. Hereditary cancer predisposition typically follows an autosomal dominant inheritance pattern and may be suspected based on clinical warning signs such as early disease onset, multiple primary malignancies, a strong family history, or the presence of microsatellite instability in tumour tissue. In addition to Lynch syndrome, rarer genetic conditions—including Cowden syndrome (PTEN), Li–Fraumeni syndrome (TP53), polymerase proofreading–associated polyposis (POLE/POLD1), and hereditary breast and ovarian cancer syndromes (BRCA1/2)—also contribute to hereditary endometrial cancer risk. Recognition of these genetic backgrounds is essential for accurate diagnosis, personalised surveillance, and the implementation of targeted preventive and therapeutic strategies. Despite major advances in molecular diagnostics, hereditary endometrial cancer remains frequently underdiagnosed, leading to missed opportunities for cancer prevention among affected individuals and their families. This comprehensive review summarises current evidence on hereditary predispositions to endometrial cancer, with a particular emphasis on Lynch syndrome, and discusses underlying genetic mechanisms, inheritance patterns, diagnostic strategies, and clinical implications for screening, genetic counselling, and treatment optimisation.

## 1. Introduction

Endometrial cancer is among the most common gynaecologic malignancies in high-income countries. Its development is strongly influenced by factors that prolong oestrogen exposure without the counterbalancing effect of progesterone, including obesity and the use of oestrogen-only hormone replacement therapy [1]. Although the majority of cases are sporadic and driven by hormonal and metabolic factors, a clinically relevant subset arises in the context of hereditary cancer syndromes. Among these, Lynch syndrome is the most prominent entity, accounting for approximately 3% of all endometrial cancers [2,3].

Hereditary cancer syndromes are estimated to contribute to approximately 10% of all malignancies, yet they remain frequently underdiagnosed in routine clinical practice. These syndromes result from germline pathogenic variants that substantially increase cancer susceptibility, most commonly following an autosomal dominant inheritance pattern, with a 50% transmission risk to offspring [4]. In clinical settings, recognition is often prompted by early-onset disease, multiple primary tumours, strong family history, or atypical cancer presentations. Among inherited cancer predisposition syndromes, Lynch syndrome and hereditary breast and ovarian cancer (HBOC) are the most prevalent [5]. In this context, the 2024 NCCN Guidelines for Genetic/Familial High-Risk Assessment: Colorectal, Endometrial, and Gastric emphasise the expanding role of systematic germline testing and multigene panel analysis in patients with endometrial cancer, moving beyond traditional colorectal-centred screening strategies [6].

Accumulating evidence from molecular pathology, immunogenomics, and multi-omics profiling indicates that Lynch syndrome–associated endometrial cancer represents a biologically distinct disease entity rather than merely a hereditary analogue of sporadic tumours. It is characterised by DNA mismatch repair (MMR) deficiency, microsatellite instability (MSI), and a hypermutated, immune-inflamed tumour microenvironment [7,8,9]. These molecular features have direct clinical relevance, influencing tumour classification, prognostication, and therapeutic decision-making, particularly with respect to immune checkpoint inhibitor therapy targeting the PD-1/PD-L1 axis [10,11].

Reflecting this paradigm shift, recent international guidelines have formally integrated molecular classification into the diagnostic and staging framework of endometrial cancer. The 2023 FIGO staging update incorporates molecular subgroups (POLEmut, MMRd, p53abn, and NSMP), underscoring the clinical value of genomic stratification [12]. Similarly, the 2025 ESGO–ESTRO–ESP guidelines recommend universal molecular profiling as part of the standard diagnostic work-up and management of endometrial carcinoma [13].

The aim of this review is to provide a clinically oriented synthesis of hereditary predispositions to endometrial cancer, with a particular focus on Lynch syndrome. By integrating current knowledge on genetic mechanisms, molecular classification, and guideline-based diagnostic and therapeutic strategies, this review highlights how combined molecular, pathological, and clinical assessment can improve patient stratification, inform genetic counselling, and support precision oncology approaches in endometrial cancer.

## 2. Hereditary Endometrial Cancer—General Characteristics

### 2.1. Frequency of Hereditary Cases of Endometrial Cancer

Endometrial cancer is predominantly sporadic; however, approximately 2–5% of cases arise in the context of well-defined hereditary cancer syndromes, most notably Lynch syndrome [14,15]. Lynch syndrome, caused by germline mutations in DNA mismatch repair (MMR) genes, accounts for approximately 2–3% of all endometrial cancer cases [3,15].

Data from the Prospective Lynch Syndrome Database (PLSD) indicate that the lifetime risk of endometrial cancer among carriers of pathogenic MMR gene variants—MLH1 (MutL homolog 1), MSH2 (MutS homolog 2), MSH6 (MutS homolog 6), and PMS2 (postmeiotic segregation increased 2)—varies by gene, ranging from approximately 40–60% and reaching up to 71% in MSH6 mutation carriers [3,14,16,17]. In high-risk populations, particularly those with a strong family history and additional Lynch syndrome–associated malignancies, the prevalence may be even higher [3].

### 2.2. Indications for Suspecting a Hereditary Nature of the Disease (Age, Family History, Histological Features)

Not every patient with endometrial cancer should be routinely suspected of a hereditary predisposition; however, several clinical warning signs may indicate an underlying genetic cause:

- diagnosis at a young age, particularly before 50 years;
- a history of endometrial cancer, colorectal cancer, or other Lynch syndrome–associated malignancies in first- or second-degree relatives;
- characteristic histopathological features, such as microsatellite instability (MSI-H) or loss of MMR protein expression;
- the presence of multiple primary cancers in the same patient, either synchronous or metachronous [16,18,19,20].

Given that some hereditary cases may otherwise remain undetected, an increasing number of professional societies now recommend universal screening of all endometrial cancers for MMR deficiency or MSI [18].

## 3. Lynch Syndrome—Main Hereditary Predisposition

### 3.1. Cancer Spectrum and Risk Profile in Lynch Syndrome

Lynch syndrome is associated with a markedly increased lifetime risk of multiple malignancies, most prominently colorectal and endometrial cancer, but also ovarian and gastric cancer. This broad cancer spectrum reflects the systemic nature of mismatch repair (MMR) deficiency and underpins the clinical importance of Lynch syndrome across several oncologic disciplines, including gynaecologic oncology [21].

Colorectal cancer represents the most frequent malignancy in individuals with Lynch syndrome, with estimated lifetime risks ranging from approximately 40% to 80%, depending on sex and the specific pathogenic variant involved [21]. The disease typically develops at a substantially younger age than in the general population, most often between 40 and 50 years, and shows a predilection for the proximal colon [22,23,24]. These features form the basis for intensive colonoscopic surveillance strategies and early screening initiation in mutation carriers.

In women, endometrial cancer constitutes the second most common malignancy associated with Lynch syndrome and, in many cases, may represent the sentinel cancer leading to the diagnosis of the syndrome within a family [25,26]. The estimated lifetime risk of endometrial cancer ranges from 30% to 60%, with several studies reporting risks comparable to or exceeding those of colorectal cancer in female mutation carriers [25]. Endometrial cancer in Lynch syndrome occurs earlier than sporadic disease, with a median age at diagnosis up to a decade younger, and displays gene-dependent variability in risk, being highest in carriers of *MLH1* mutations and lower in those with *MSH6* or *PMS2* variants [27].

The risk of ovarian cancer is also increased in women with Lynch syndrome, although to a lesser extent than colorectal or endometrial cancer. Lifetime risk estimates range from approximately 4% to 12%, with ovarian tumours typically presenting at a younger age than in the general population, often in the early to mid-forties [17,28,29]. While less frequent, this elevated risk has important implications for surveillance and risk-reducing strategies.

Gastric cancer represents another component of the Lynch syndrome cancer spectrum, with reported lifetime risks between 6% and 13% and a mean age at diagnosis of approximately 56 years [30]. The clinical relevance of gastric cancer varies geographically, being particularly significant in regions with high background incidence, such as East Asia, which may justify region-specific surveillance approaches [31].

Collectively, the increased and early-onset risk of colorectal, endometrial, ovarian, and gastric cancers defines Lynch syndrome as a prototypical multi-organ cancer predisposition syndrome. Understanding this cancer spectrum is essential for guiding personalised surveillance, preventive strategies, and multidisciplinary management in affected individuals and their families.

### 3.2. Genetic and Molecular Basis of Lynch Syndrome

Lynch syndrome arises from germline pathogenic variants affecting the DNA mismatch repair (MMR) system, a key mechanism responsible for maintaining genomic stability during DNA replication. These variants are inherited predominantly in an autosomal dominant manner and confer a markedly increased lifetime risk of malignancy through the accumulation of replication-associated errors. The molecular pathogenesis of Lynch syndrome follows the classical Knudson two-hit model, in which a germline alteration constitutes the first hit and subsequent somatic inactivation of the remaining wild-type allele results in biallelic MMR deficiency, microsatellite instability (MSI), and tumorigenesis [32,33,34,35,36,37,38,39].

The core genes implicated in Lynch syndrome include *MLH1*, *MSH2*, *MSH6*, and *PMS2*, with additional cases arising from deletions involving the *EPCAM* gene that lead to secondary epigenetic silencing of *MSH2*.

Although pathogenic variants in *MLH1* and *MSH2* account for the majority of cases and are generally associated with higher cancer penetrance, variants in *MSH6* and *PMS2* tend to present with later onset and lower overall cancer risk, while maintaining a relatively high proportion of endometrial cancer among affected women. Gene-specific differences in endometrial cancer risk and age at onset have been quantified in large prospective cohorts, including the Prospective Lynch Syndrome Database, as summarised in Table 1 [21,40].

**Figure 1:**
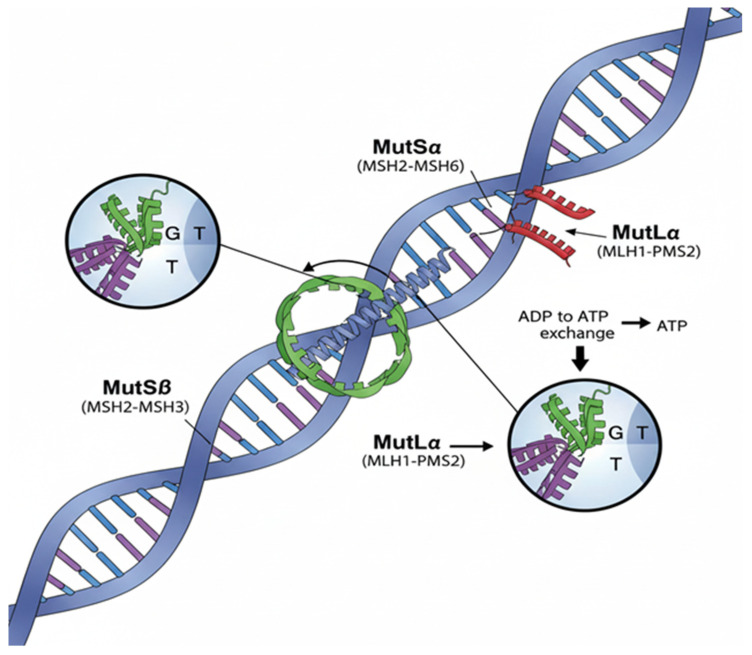
Mechanism of action of MMR proteins.

At the molecular level, MMR deficiency disrupts the accurate correction of base–base mismatches and insertion–deletion loops generated during DNA replication. Under physiological conditions, these errors are recognised by the MutSα (*MSH2–MSH6*) or MutSβ (*MSH2–MSH3*) complexes and subsequently processed by the MutLα (*MLH1–PMS2*) complex, which coordinates downstream repair events (Figure 1, Table 2) [44,45].

**Figure 2:**
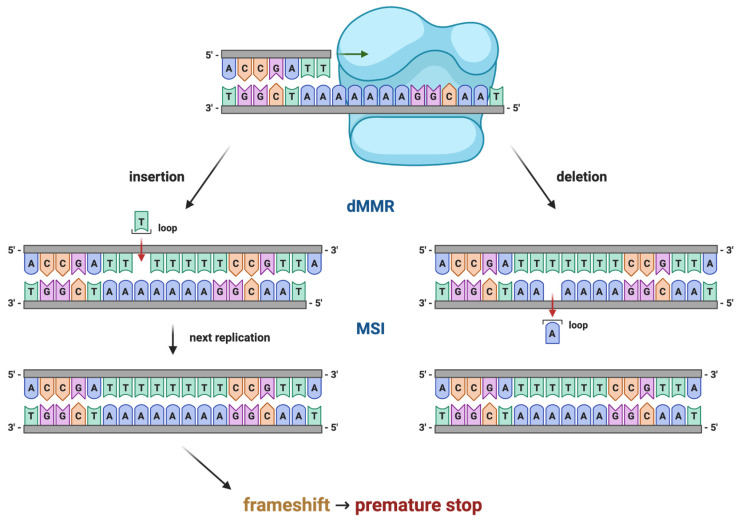
dMMR causes MSI, and non-triplet coding indels trigger frameshifts that generate premature termination codons.

Loss of function of any of these components leads to persistence of replication errors, particularly within microsatellite regions, resulting in the MSI phenotype characteristic of Lynch syndrome–associated tumours (Figure 2).

In addition to sequence variants, epigenetic mechanisms contribute to the molecular heterogeneity of Lynch syndrome and Lynch-like phenotypes. Constitutional *MLH1* epimutation, caused by promoter hypermethylation of one *MLH1* allele, results in allele-specific transcriptional silencing and loss of *MLH1/PMS2* expression on immunohistochemistry. Similarly, deletions affecting the 3′ end of *EPCAM* induce transcriptional read-through and tissue-specific hypermethylation of the adjacent *MSH2* promoter, leading to functional MMR deficiency despite the absence of a germline *MSH2* sequence variant [46,47,48,49,50,51]. These epigenetic alterations may be mosaic or tissue-restricted, complicating detection by standard germline assays and posing significant diagnostic challenges.

Beyond classical Lynch syndrome, defects in DNA replication fidelity arising from germline mutations in other genes may confer overlapping molecular features. Polymerase proofreading–associated polyposis, caused by pathogenic variants in the exonuclease domains of *POLE* or *POLD1*, results in an ultra-mutated genomic phenotype with a high tumour mutational burden and pronounced immune infiltration. Although typically microsatellite stable, these tumours share important biological and therapeutic characteristics with MMR-deficient cancers, including favourable prognosis and potential sensitivity to immunotherapy. Additionally, hereditary cancer syndromes, such as phosphatase and tensin homolog hamartoma tumour syndrome (*PTEN*), Peutz–Jeghers syndrome (*STK11*), and Li–Fraumeni syndrome (*TP53*), are also associated with increased endometrial cancer risk, albeit with lower penetrance compared with Lynch syndrome.

Collectively, the genetic and molecular landscape of Lynch syndrome encompasses a spectrum of germline and epigenetic alterations that converge on defective DNA repair and genomic instability. Integration of molecular mechanisms with gene-specific risk profiles and immunobiological consequences provides a framework for accurate diagnosis, refined risk assessment, and the development of personalised surveillance and treatment strategies in hereditary endometrial cancer.

The DNA mismatch repair pathway is initiated by the recognition of base–base mismatches or insertion–deletion loops generated during DNA replication. The MutSα complex (MSH2–MSH6) primarily recognizes single-base mismatches and small insertion–deletion loops, whereas MutSβ (MSH2–MSH3) preferentially binds larger insertion–deletion loops. Upon mismatch recognition, ATP binding induces a conformational change in MutS, allowing the recruitment of the MutLα complex (MLH1–PMS2). MutLα acts as a molecular coordinator, activating downstream repair events including strand discrimination, endonuclease activity, and recruitment of exonucleases and DNA polymerases. This coordinated process leads to excision of the error-containing DNA strand and accurate resynthesis, thereby maintaining genomic stability. Germline defects in MMR components disrupt this pathway, resulting in microsatellite instability and increased cancer susceptibility, as observed in Lynch syndrome.

### 3.3. Causes of Mismatch Repair Deficiency in Endometrial Cancer

Mismatch repair deficiency (dMMR) in endometrial cancer represents a biologically heterogeneous phenomenon arising from distinct genetic and epigenetic mechanisms. Although dMMR is a defining molecular feature of Lynch syndrome–associated tumours, it may also occur in sporadic cancers or in so-called Lynch-like cases, each with different diagnostic, prognostic, and clinical implications [52,53,54,55]. Distinguishing among these causes is essential for appropriate patient management, genetic counselling, and family risk assessment.

In hereditary endometrial cancer associated with Lynch syndrome, dMMR results from a germline pathogenic variant in one of the MMR genes (*MLH1*, *MSH2*, *MSH6*, or *PMS2*) followed by somatic inactivation of the remaining allele, consistent with the Knudson two-hit model [39,56]. This leads to complete loss of MMR function, high levels of microsatellite instability, and a hypermutated tumour phenotype [36,37,38]. Immunohistochemistry (IHC) typically demonstrates loss of the corresponding MMR protein(s) in tumour tissue, following characteristic heterodimeric patterns (e.g., concurrent loss of MLH1 and PMS2 or MSH2 and MSH6) [57]. These tumours frequently arise at a younger age and are associated with an increased risk of synchronous and metachronous malignancies [21,40].

In contrast, the most common cause of dMMR in endometrial cancer overall is sporadic epigenetic silencing of *MLH1* through promoter hypermethylation [30,49]. This mechanism leads to loss of MLH1 and PMS2 protein expression on IHC but is not associated with an inherited cancer predisposition [49,50,51]. Sporadic *MLH1*-hypermethylated tumours typically occur in older patients, often in the context of obesity and metabolic risk factors, and lack a significant family history of Lynch syndrome–associated cancers [30,31]. Testing for *MLH1* promoter methylation is therefore a critical step in the diagnostic algorithm to differentiate sporadic dMMR tumours from true Lynch syndrome–related cases [49,50].

A diagnostically challenging subgroup is represented by Lynch-like tumours, which exhibit dMMR and/or MSI in the absence of detectable germline MMR gene mutations or *MLH1* promoter hypermethylation [46,47]. Increasing evidence suggests that many of these cases result from biallelic somatic inactivation of MMR genes, including combinations of somatic point mutations, small insertions or deletions, and loss of heterozygosity [58,59]. Although these tumours share molecular and immunological features with Lynch syndrome–associated cancers, they do not confer an inherited cancer risk, highlighting the importance of tumour–normal paired sequencing for accurate classification [58].

Additional mechanisms contributing to apparent dMMR include technical and interpretative limitations of diagnostic assays. Pre-analytical variables, antibody selection, tumour heterogeneity, and equivocal staining patterns may complicate IHC interpretation [52,57]. Similarly, MSI testing may yield discordant results depending on the marker panel used, tumour cellularity, and analytical thresholds [53]. The increasing use of next-generation sequencing has also led to the identification of variants of uncertain significance, which require cautious interpretation in a multidisciplinary clinical context [60].

From a therapeutic perspective, the underlying cause of dMMR has important implications. While dMMR/MSI-high endometrial cancers—regardless of aetiology—are generally associated with increased tumour mutational burden and enhanced responsiveness to immune checkpoint inhibitors [53], only patients with confirmed Lynch syndrome require intensified cancer surveillance, risk-reducing strategies, and cascade testing of at-risk relatives [21,32,33,34]. Consequently, a stepwise diagnostic approach integrating IHC, MSI testing, *MLH1* promoter methylation analysis, and germline and somatic genetic testing is essential for accurate tumour classification and optimal clinical decision-making [50,52,53,54,55].

### 3.4. Clinical Characteristics of Endometrial Cancer in Lynch Syndrome

Endometrial cancer associated with Lynch syndrome exhibits distinct clinical and pathological characteristics that differentiate it from sporadic disease and may facilitate identification of patients at increased hereditary cancer risk [61,62,63]. These features relate primarily to age at onset, tumour location, histological spectrum, and the risk of synchronous or metachronous malignancies, as summarised in Table 3.

One of the most prominent clinical hallmarks of Lynch syndrome–associated endometrial cancer is its earlier age at diagnosis. While sporadic endometrial cancer is typically diagnosed around the age of 60 years, women with Lynch syndrome develop the disease approximately 10–15 years earlier, with a median age of onset of about 49 years [61]. Age at diagnosis varies according to the affected MMR gene: carriers of *MLH1* and *MSH2* mutations tend to develop endometrial cancer earlier, often between 39 and 49 years, whereas *MSH6* mutation carriers are frequently diagnosed later, usually between 50 and 60 years of age [61]. Tumours associated with *PMS2* mutations generally present at the oldest age and show lower overall penetrance [21,40].

Tumour location within the uterus represents another distinguishing clinical feature. Lynch syndrome–associated endometrial cancers demonstrate a significantly higher frequency of involvement of the lower uterine segment (LUS), reported in approximately 14–30% of cases, compared with only 3–5% in sporadic endometrial cancer [61,62]. LUS involvement is clinically relevant, as it has been associated with an increased risk of lymph node metastases and less favourable outcomes, underscoring the need for careful surgical staging in this subgroup [62].

With regard to histopathology, the majority of Lynch syndrome–associated endometrial cancers are of the endometrioid type, accounting for approximately 70–90% of cases [62]. These tumours are often low to intermediate grade; however, compared with sporadic endometrial cancer, Lynch syndrome is associated with a relatively higher proportion of non-endometrioid histologies, including serous and clear cell carcinomas, particularly among carriers of *MSH2* mutations [61]. Moreover, a substantial subset of cases presents with high-grade (G3) tumours, highlighting the importance of comprehensive pathological evaluation and molecular classification [62,63].

Patients with Lynch syndrome are also characterised by an increased risk of synchronous and metachronous primary malignancies. Following a diagnosis of endometrial cancer, the cumulative risk of developing a second primary tumour—most commonly colorectal, ovarian, or urinary tract cancer—has been estimated to range from 11% to 33% [21,63]. This elevated risk has significant implications for long-term surveillance strategies and supports the role of risk-reducing surgery, including prophylactic hysterectomy and bilateral salpingo-oophorectomy, in selected patients who have completed childbearing [32,33,34].

Collectively, the clinical phenotype of Lynch syndrome–associated endometrial cancer—characterised by early onset, preferential involvement of the lower uterine segment, distinct histopathological features, and a high risk of additional primary malignancies—provides important diagnostic clues and directly informs surveillance, surgical management, and genetic counselling [21,61,62,63].

These clinical and molecular distinctions provide the rationale for tailored diagnostic algorithms and universal molecular screening strategies in endometrial cancer, which are discussed in the following sections.

## 4. Other Hereditary Syndromes Related to Endometrial Cancer

### 4.1. Cowden Syndrome (PTEN Mutations)

Cowden syndrome (CS) is a rare autosomal dominant genodermatosis [64]. It is caused by germline mutations in the PTEN tumour suppressor gene and is associated with an increased risk of multisystem hamartomas and malignancies [64,65]. The *PTEN* gene is located on the long arm of chromosome 10 (10q22–23) and encodes a lipid phosphatase that negatively regulates the PI3K/AKT signalling pathway. Loss of PTEN function leads to dysregulated cell survival, proliferation, and tumour development [66,67].

Cowden syndrome belongs to the spectrum of PTEN hamartoma tumour syndromes (PHTS), which also includes Bannayan–Riley–Ruvalcaba syndrome, PTEN-related Proteus syndrome, and PTEN-related Proteus-like syndrome [68]. Clinically, CS is characterized by multiple mucocutaneous hamartomas, including trichilemmomas, as well as gastrointestinal polyps [69,70]. Individuals with Cowden syndrome are at increased risk of several malignancies, most notably breast cancer in both women and men, as well as thyroid, renal, and endometrial cancers [68,71].

#### 4.1.1. Endometrial Cancer in Cowden Syndrome

Endometrial cancer is a recognised core component of Cowden syndrome (CS). The reported lifetime risk of endometrial cancer among patients with germline *PTEN* mutations varies across studies, ranging from 13 to 19% [72] to as high as 28.2% [73]. Endometrial cancer in CS is frequently diagnosed at a young age, often before 50 years. Notably, extremely early-onset cases have been described, including reports of adolescents with CS [74,75].

In this exceptional case, the patient developed grade 1 endometrial adenocarcinoma at the age of 14, shortly after the onset of fibrocystic breast disease and the detection of colonic polyps; a definitive diagnosis of Cowden syndrome was established at the age of 20 [74].

#### 4.1.2. Diagnostic Criteria

The diagnostic criteria for Cowden syndrome have been established by the National Comprehensive Cancer Network (NCCN). A diagnosis of Cowden syndrome can be made if one of the following conditions is met:

- the presence of at least three major criteria;
- a combination of two major criteria and at least three minor criteria.

In individuals from families with a known PTEN mutation, Cowden syndrome or phosphatase and tensin homolog hamartoma tumour syndrome (PHTS) may be diagnosed if any of the following criteria are fulfilled:

- two or more major criteria;
- one major criterion and at least two minor criteria;
- a total of at least three minor criteria.

The major and minor diagnostic criteria are summarised in Table 4.

Although not yet formally validated, surveillance recommendations developed for Lynch syndrome have also been proposed for patients with Cowden syndrome. These include annual endometrial biopsy starting between 30 and 35 years of age, or five years earlier than the youngest age at diagnosis of endometrial cancer in the family. In postmenopausal women, annual transvaginal ultrasound with biopsy of any suspicious findings has also been suggested [72].

### 4.2. Li-Fraumeni Syndrome (TP53 Mutations)

Li-Fraumeni syndrome (LFS) is a rare autosomal dominant cancer predisposition syndrome caused by germline pathogenic variants in the TP53 tumour suppressor gene, resulting in a markedly increased lifetime cancer risk [76,77,78,79]. The tumour spectrum of LFS is dominated by sarcomas, premenopausal breast cancer, brain tumours, adrenocortical carcinoma, and leukaemia, typically characterised by early onset and a high incidence of multiple primary malignancies [79,80,81].

Endometrial cancer has been reported only sporadically in TP53 mutation carriers and does not constitute a core component of the LFS tumour spectrum. Available evidence is limited to isolated case reports and small family series, without population-based risk estimates or dedicated studies evaluating endometrial cancer risk in LFS [82,83,84]. Consequently, routine endometrial cancer surveillance is not currently recommended for TP53 mutation carriers, and LFS should be considered only in selected differential diagnostic scenarios, particularly in patients with very early-onset disease or multiple malignancies.

### 4.3. Hereditary Ovarian and Breast Cancer Syndromes (e.g., BRCA1/2)

Hereditary breast and ovarian cancer (HBOC) syndrome, caused by germline pathogenic variants in *BRCA1* and *BRCA2*, is primarily associated with a markedly increased risk of breast and ovarian cancers due to impaired homologous recombination–mediated DNA repair [85,86,87,88,89]. The association between *BRCA1/2* mutations and endometrial cancer risk remains controversial.

Some studies have reported a modestly increased risk of endometrial cancer—particularly the aggressive serous subtype-in *BRCA1* mutation carriers, whereas other large cohort analyses have failed to demonstrate a consistent association [90,91,92,93,94,95,96,97,98]. Overall, endometrial cancer represents a relatively uncommon malignancy in HBOC, and current evidence does not support routine prophylactic hysterectomy solely for endometrial cancer prevention in *BRCA1/2* carriers. Nevertheless, hereditary cancer syndromes, including HBOC, should be considered in patients with endometrial cancer and a strong personal or family history of breast or ovarian cancer, particularly when tumour histology suggests a serous phenotype [99,100].

### 4.4. Rare Germline Mutations with Potential Significance

Although Lynch syndrome classically results from germline loss-of-function variants in mismatch repair genes, rare germline defects in DNA replicative polymerases-most notably POLE and POLD1—have increasingly been recognized as biologically and clinically relevant overlaps or mimics of Lynch syndrome. Germline exonuclease-domain (proofreading) variants in POLE/POLD1 cause polymerase proofreading–associated polyposis, characterized by a high tumour mutational burden and a strong predisposition to colorectal and endometrial cancers. These variants may give rise to ultramutated tumours and, in some cases, co-occur with mismatch repair deficiency, creating so-called “*POLE–Lynch*” or multilocus phenotypes that complicate diagnosis and clinical management [101,102].

Case reports and family-based studies have documented both clearly pathogenic germline POLE variants-such as exonuclease-domain mutations associated with multiple primary tumours and polyposis-and collision cases in which a pathogenic POLE variant coexists with a pathogenic MMR gene variant. These rare *MINAS* (“*POLE–LYNCH*”) *collision* scenarios may result in very early-onset, hypermutated malignancies and, in selected cases, striking responses to immune checkpoint blockade [103,104].

Importantly, many non-truncating exonuclease-domain substitutions in POLE and POLD1 remain classified as variants of uncertain significance (VUS). Recent efforts applying gene-specific ACMG/AMP criteria have enabled reclassification of a subset of these variants (17 pathogenic/likely pathogenic and 17 benign/likely benign among 128 curated exonuclease-domain missense variants); however, a substantial proportion of VUS persists and requires integration of tumour mutational signatures, co-segregation analyses, functional assays, and population-level data for definitive interpretation [102].

Functional and structural studies—including yeast-based mutagenesis models and biochemical proofreading assays-together with focused family investigations have proven instrumental in reclassifying specific POLE variants, such as p.Tyr458Phe and p.Thr278Lys, as pathogenic. These examples illustrate how multidisciplinary evaluation can substantially alter clinical interpretation, surveillance recommendations, and cascade testing strategies for affected families [105,106].

Clinicians should therefore consider POLE/POLD1 testing in patients with unexplained suspected Lynch syndrome or familial colorectal cancer and polyposis. Non-truncating exonuclease-domain variants should be interpreted using gene-specific criteria and, whenever possible, complemented by tumour mutational profiling and functional data. Accurate classification is critical, as misinterpretation has direct implications for cancer surveillance, risk assessment of relatives, and therapeutic decision-making, including eligibility for immunotherapy [103,107].

## 5. Diagnostics of Hereditary Endometrial Cancer

The diagnosis of hereditary endometrial cancer relies on an integrated, multistep approach combining tumour-based molecular screening with confirmatory germline genetic testing [108]. Current international guidelines issued by ESGO, NCCN, and ESMO recommend universal screening for mismatch repair (MMR) deficiency in all patients with newly diagnosed endometrial cancer, regardless of age or family history, to facilitate identification of hereditary cancer predisposition and guide therapeutic decision-making [6,109,110].

**Figure 3:**
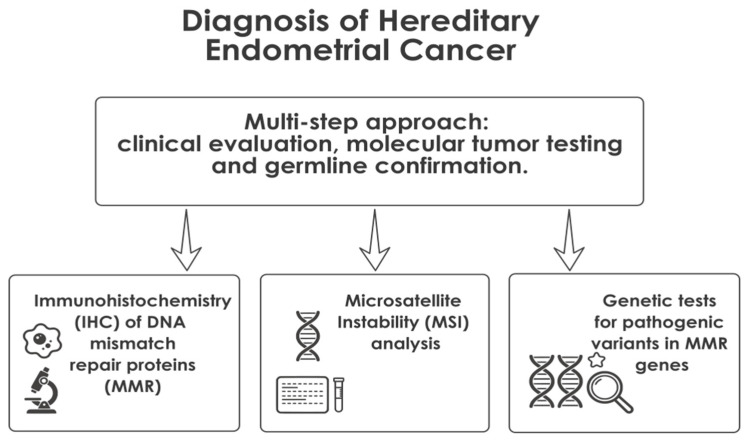
The three pillars of Lynch syndrome diagnosis.

From a practical perspective, diagnostic evaluation is based on three complementary pillars:

(i) immunohistochemical (IHC) assessment of MMR protein expression,
(ii) microsatellite instability (MSI) testing, and
(iii) germline genetic testing for pathogenic variants in MMR genes (Figure 3) [111,112].

### 5.1. MMR Protein Immunohistochemistry as a Screening Tool

Immunohistochemical evaluation of MMR protein expression is the most widely used first-line screening method for identifying mismatch repair deficiency in endometrial cancer. Universal IHC screening is currently recommended by major international guidelines, as it enables efficient identification of patients who may benefit from further molecular analyses and genetic counselling [6,109,110].

MMR proteins function as heterodimeric complexes: MLH1 pairs with PMS2 (MutLα), while MSH2 pairs with MSH6 (MutSα). Loss of a dominant partner (MLH1 or MSH2) typically leads to secondary degradation of the corresponding binding partner, whereas isolated loss of PMS2 or MSH6 usually reflects a primary alteration in the respective gene [113,114].

Characteristic IHC staining patterns and their molecular and clinical implications are summarized in Table 5. These patterns allow differentiation between sporadic tumours, most commonly associated with MLH1 promoter hypermethylation, and cases suspicious for Lynch syndrome, particularly those showing loss of MSH2/MSH6 or isolated loss of PMS2 or MSH6 [114,115].

Multiple studies have demonstrated high concordance between IHC and MSI testing, with reported sensitivity and specificity of approximately 95–96%, supporting the use of IHC as a reliable surrogate for MSI analysis in routine diagnostics [115,116]. Simplified two-antibody panels (PMS2 and MSH6) have shown diagnostic performance comparable to the full four-antibody panel and may represent a cost-effective alternative in selected settings [116,117,118].

Overall, MMR protein immunohistochemistry provides rapid, robust, and clinically actionable information and remains a cornerstone of diagnostic algorithms for hereditary endometrial cancer [112,119].

**Table 5:** Patterns of MMR Protein Loss in Endometrial Cancer and Their Molecular Implications.

IHC Pattern	Underlying Molecular Mechanism	Clinical/Genetic Interpretation	Recent Insights (2023–2025)	Key References
Loss of MLH1/PMS2	MLH1 inactivation (promoter methylation or germline variant) causing secondary PMS2 loss	Usually sporadic EC (MLH1 methylation); unmethylated cases suggest Lynch syndrome (MLH1	MLH1-methylated EC represents an epigenetically distinct subtype with poorer prognosis and reduced ICI response compared with LS-associated dMMR	[111,112,115,120,121,122]
Loss of MSH2/MSH6	MSH2 loss destabilizes MSH6 (MutSα complex); may involve EPCAM deletion	Typical of Lynch syndrome (MSH2); EPCAM deletions cause MSH2 silencing	Highly immune-inflamed phenotype with strong IFN-γ signaling and favorable ICI response	[117,123]
Isolated loss of MSH6	Germline or somatic MSH6 mutation; preserved MSH2	Lynch syndrome (MSH6); later onset, endometrioid histology, lower MSI	Hypermutated but immune-active EC with good PD-1 inhibitor outcomes	[113,114,124]
Isolated loss of PMS2	PMS2 mutation with intact MLH1; technical detection challenges	Lynch syndrome (PMS2); low penetrance, diagnostic complexity	Lower TMB and intermediate immune activation; improved detection via pseudogene-aware assays	[41,115,116]
Intact MMR expression	Proficient MMR (pMMR/MSI-stable)	Sporadic EC; usually not eligible for ICI monotherapy	Subset shows POLE mutations or high TMB, conferring immunogenic potent	[120,125]

These characteristic staining patterns enable differentiation between sporadic tumours—most commonly associated with MLH1 promoter hypermethylation—and cases suspicious for Lynch syndrome, particularly those demonstrating loss of MSH2/MSH6 or isolated loss of PMS2 or MSH6 [114,115].

Multiple studies have demonstrated a high concordance between immunohistochemistry and molecular testing for mismatch repair deficiency in endometrial cancer, with reported sensitivity and specificity of approximately 95–96%, supporting the use of IHC as a reliable surrogate for microsatellite instability testing [115,116]. Accordingly, international guidelines recommend universal IHC screening in all newly diagnosed endometrial carcinomas to identify patients who require further molecular analyses or germline genetic testing [110,126,127].

Simplified screening strategies using a two-antibody panel (PMS2 and MSH6) have demonstrated diagnostic performance comparable to that of the full four-antibody panel, providing a cost-effective alternative without compromising diagnostic accuracy [116,117,118]. Overall, MMR protein immunohistochemistry remains a cornerstone of endometrial cancer diagnostics, delivering rapid and clinically actionable information for patient stratification and assessment of hereditary cancer risk [112,119].

### 5.2. MSI Testing

Microsatellite instability (MSI) results from defective mismatch repair and represents a key molecular hallmark of Lynch syndrome-associated endometrial cancer. MSI testing compares microsatellite length variations between tumour and normal DNA, most commonly using PCR-based panels [128].

MSI results are classified as MSI-high (MSI-H), MSI-low (MSI-L), or microsatellite stable (MSS). MSI-H status is typical of Lynch syndrome-associated tumours but also occurs in sporadic endometrial cancers, most often due to MLH1 promoter hypermethylation [26,41].

High concordance between MSI testing and MMR IHC has been reported, and the combined use of both methods is considered the diagnostic gold standard for Lynch syndrome screening in endometrial cancer [26]. MSI testing is particularly valuable in cases with equivocal IHC results or discordant findings.

Clinically, MSI-H status has important prognostic and therapeutic implications. MSI-H endometrial cancers, especially of the endometrioid subtype, are associated with improved response to immune checkpoint inhibitors, including pembrolizumab and dostarlimab, particularly in advanced or recurrent disease [11,129].

### 5.3. Genetic Testing

Germline testing for pathogenic variants in MMR genes is essential for confirmation of hereditary endometrial cancer and for subsequent clinical management. While Sanger sequencing was historically considered the gold standard, next-generation sequencing (NGS) has become the preferred first-line approach due to its higher throughput, broader coverage, and ability to analyse multiple cancer predisposition genes simultaneously [130,131,132].

NGS-based multigene panels routinely include MLH1, MSH2, MSH6, PMS2, and EPCAM, and often extend to additional genes associated with endometrial cancer susceptibility. Germline testing is typically performed on DNA derived from peripheral blood, with variant interpretation conducted according to established clinical genetics guidelines [108,133].

Pathogenic germline MMR variants are identified in approximately 4.5–23% of patients with endometrial cancer, depending on population characteristics and testing strategy, with Lynch syndrome accounting for the majority of hereditary cases [133]. When a pathogenic variant is detected, referral for genetic counselling and implementation of syndrome-specific surveillance strategies are mandatory [6,21,134].

Despite its advantages, NGS testing presents challenges, including detection of variants of uncertain significance, technical difficulties related to pseudogenes (e.g., PMS2), and limitations in identifying large genomic rearrangements. Complementary methods such as MLPA or long-range PCR may therefore be required in selected cases [130,135].

Emerging technologies, including whole-genome sequencing and CRISPR-based diagnostic platforms, may further enhance diagnostic accuracy in the future but currently remain largely investigational [136,137].

### 5.4. Diagnostic Criteria

Several clinical and molecular criteria have been developed to identify patients at increased risk of Lynch syndrome and to guide referral for genetic counselling and germline testing. Although historically based on family history and tumour characteristics, these criteria are now increasingly complemented by universal molecular screening strategies in endometrial cancer [138,139,140,141].

#### 5.4.1. Diagnostic Guidelines

Amsterdam II criteria were published in 1999 as an extension of the original Amsterdam I criteria introduced in 1991 [138,139]. Although the Amsterdam II criteria demonstrate high specificity (up to 98%), their sensitivity remains limited (27–42%), which may result in missed diagnoses—particularly in patients in whom endometrial cancer is the first or only manifestation of Lynch syndrome –associated malignancy [140,141].

To improve diagnostic sensitivity, the Bethesda guidelines were introduced in 1997 and subsequently revised in 2004. These guidelines were designed to identify individuals who should undergo testing for microsatellite instability (MSI), a molecular hallmark of mismatch repair (MMR) deficiency in Lynch syndrome [20]. The Bethesda guidelines incorporate broader clinical and pathological criteria and are particularly useful for selecting tumours for molecular testing. A summary of the Amsterdam II criteria and the revised Bethesda guidelines is provided in Table 6.

#### 5.4.2. Indications for Referral to a Genetic Counselling Centre

With the increasing implementation of universal screening for mismatch repair (MMR) deficiency in endometrial cancer, referral to genetic counselling is now guided by an integrated assessment of clinical, pathological, and molecular findings [144]. Evaluation for Lynch syndrome should begin with a detailed family cancer history spanning at least three generations and including first-, second-, and third-degree relatives [112]. All malignancies should be documented, along with the age at diagnosis, whenever possible.

Genetic testing for Lynch syndrome should be considered in patients who meet one or more of the following criteria:

- Fulfilment of the Amsterdam II criteria [139]
- Fulfilment of the revised Bethesda guidelines [20]
- Diagnosis of endometrial cancer before the age of 50 years
- Known Lynch syndrome in the family [6]
- Estimated Lynch syndrome risk ≥ 5% based on predictive models such as MMRpro, PREMM, or related algorithms [145]

Genetic counselling enables comprehensive risk assessment, informed consent for genetic testing, and the implementation of appropriate surveillance and risk-reducing strategies for patients and their at-risk relatives [21,144,146].

### 5.5. Diagnostic Algorithm

**Figure 4:**
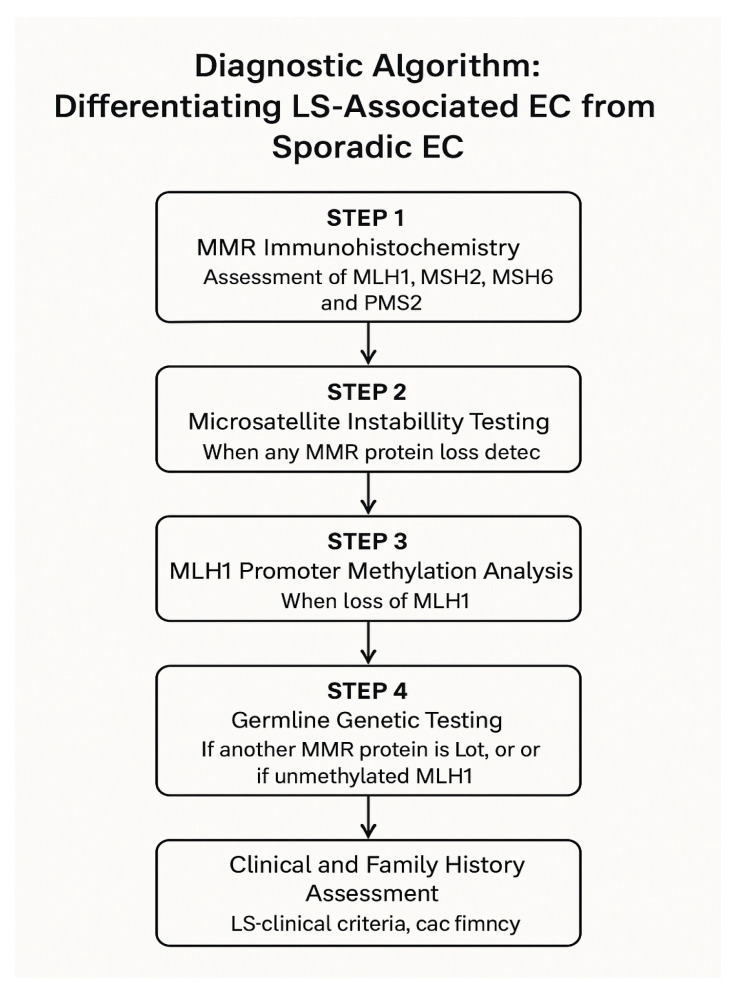
Diagnostic algorithm: Lynch syndrome-associated endometrial cancer vs. Sporadic endometrial cancer.

Current clinical practice employs a structured four-step diagnostic algorithm (Figure 4) to identify cases of endometrial cancer associated with Lynch syndrome and to distinguish them from sporadic tumours.

This standardized workflow integrates molecular, immunohistochemical, and genetic analyses, enabling accurate diagnosis, risk assessment, and appropriate management of patients and their families [6]. The diagnostic sequence begins with immunohistochemical evaluation of MMR protein expression, followed by MSI testing and MLH1 promoter methylation analysis, when loss of MLH1/PMS2 expression is detected. If hereditary MMR deficiency is suspected, confirmatory germline genetic testing is conducted. Finally, clinical and family history assessment provides the context for evaluating inherited cancer risk and guides personalized prevention strategies [147,148].

This comprehensive four-step algorithm aligns with current international recommendations, reflecting a paradigm shift from selective to universal molecular screening in endometrial cancer diagnostics [3]. Such a strategy not only facilitates the identification of patients with hereditary cancer syndromes but also has significant therapeutic implications, as tumours exhibiting dMMR or MSI-H phenotypes show high sensitivity to ICIs [149].

Based on the collected data regarding molecular, histological, and clinical differences between Lynch syndrome-associated and sporadic endometrial cancer (see Table 7), a diagnostic algorithm has been developed to streamline the clinical decision-making process and improve the identification of hereditary cancer cases (Table 3 and Table 7 collectively provide a comprehensive overview of the differences between hereditary (Lynch syndrome–associated) and sporadic endometrial cancer).

Step 1: MMR Immunohistochemistry

Assess expression of MLH1, MSH2, MSH6, and PMS2 proteins in the tumour tissue. The pattern of loss determines the next diagnostic step (Table 8).

Step 2: MSI Testing

Perform MSI testing to confirm mismatch repair deficiency (Table 9). This test can be used alongside or after IHC analysis.

Step 3: Germline Genetic Testing (if Lynch syndrome suspected)

Perform germline testing for MLH1, MSH2, MSH6, PMS2, and EPCAM genes. This is indicated if:

- MLH1/PMS2 loss with no MLH1 promoter methylation
- MSH2/MSH6/PMS2 loss by IHC
- Strong family history (Amsterdam II/Revised Bethesda criteria)

Step 4: Clinical and Family Assessment

Evaluate clinical and familial risk factors:

- Early onset endometrial cancer (<50 years)
- Synchronous or metachronous Lynch syndrome-related cancers
- Family history of colorectal, ovarian, gastric, or urinary tract cancers.

### 5.6. Diagnostic Challenges

Despite substantial progress in molecular pathology and genetic testing, the identification of Lynch syndrome and other hereditary cancer predispositions in endometrial cancer remains complex. Diagnostic difficulties arise from clinical and molecular limitations, the presence of Lynch-like tumours and discordant test results, as well as organizational and systemic barriers that affect the implementation of comprehensive diagnostic pathways [22,26,119,150,151,152,153].

#### 5.6.1. Clinical and Molecular Limitations

Traditional clinical criteria, including the Amsterdam II criteria and the revised Bethesda guidelines, were developed primarily for colorectal cancer and show limited sensitivity in patients with endometrial cancer, particularly in carriers of *MSH6* or *PMS2* pathogenic variants. These variants are often associated with later disease onset, lower penetrance, and non-informative family histories, leading to so-called “masked” cases of Lynch syndrome [22,119,150]. As summarized in Table 6, reliance on clinical criteria alone may result in underdiagnosis, supporting the growing consensus for universal molecular screening of all newly diagnosed endometrial cancers [26,119].

From a molecular perspective, immunohistochemistry (IHC) for mismatch repair (MMR) proteins remains the cornerstone of initial screening; however, interpretation may be complicated by technical artefacts, heterogeneous staining patterns, and intratumoural heterogeneity. In particular, loss of MLH1/PMS2 expression necessitates additional MLH1 promoter methylation testing to distinguish sporadic tumours from hereditary MMR deficiency (Table 5 and Table 8) [26,119,151]. Microsatellite instability (MSI) testing reliably identifies MMR-deficient tumours but lacks specificity for Lynch syndrome, as MSI-high status also occurs in sporadic endometrial cancers with MLH1 hypermethylation (Table 9) [25,26,119].

Although next-generation sequencing (NGS) has significantly expanded the diagnostic landscape, it introduces additional interpretative challenges. These include the frequent detection of variants of uncertain significance, difficulties in distinguishing germline from somatic alterations, and technical limitations related to pseudogenes or large genomic rearrangements in MMR genes [119,151,152,154]. Consequently, integrated interpretation of clinical data, tumour profiling, and complementary molecular methods (e.g., MLPA or long-range PCR) is essential for accurate diagnosis.

#### 5.6.2. Lynch-like Cases and Discordant Results

A particularly challenging diagnostic entity is represented by so-called “Lynch-like” tumours, which exhibit mismatch repair deficiency on IHC or MSI testing but lack identifiable germline pathogenic variants in MMR genes [26,119,151]. In many of these cases, biallelic somatic mutations, epigenetic alterations, or other tumour-specific mechanisms underlie the observed MMR-deficient phenotype.

Diagnostic complexity is further increased by discordant results between IHC and MSI testing, such as retained MMR protein expression in MSI-high tumours or, conversely, loss of MMR proteins in microsatellite-stable cancers. These discrepancies may reflect tumour heterogeneity, technical limitations, or alternative molecular pathways of genomic instability [26,119]. The practical implications of such discordant findings are illustrated in the diagnostic algorithm (Figure 4) and in the comparative overview of hereditary versus sporadic endometrial cancer (Table 7 and Table 8).

Recent advances in molecular classification, including the 2023 FIGO update integrating POLEmut, MMRd, p53abn, and NSMP subgroups, provide an additional framework for resolving diagnostically ambiguous cases and highlight the prognostic and therapeutic relevance of comprehensive tumour profiling [12].

#### 5.6.3. Organizational and Systemic Barriers

Beyond biological and technical challenges, real-world implementation of hereditary cancer diagnostics is frequently constrained by organizational and systemic factors. These include limited access to specialized molecular laboratories, variability in testing protocols and result interpretation, and the high cost of comprehensive genetic analyses, particularly multigene panels and copy-number variant testing [26,119,153,154].

Additional barriers include suboptimal communication between pathology, oncology, and clinical genetics services, lack of standardized referral pathways, and inconsistent availability of genetic counselling. Patient-related factors—such as reluctance to undergo genetic testing due to psychological concerns, potential insurance discrimination, or limited awareness of hereditary cancer risk—may further compromise the effectiveness of cascade testing in affected families [119,154].

Taken together, these challenges contribute to the persistent underdiagnosis of Lynch syndrome and other hereditary cancer predispositions in endometrial cancer. Addressing these barriers requires standardized diagnostic algorithms, broad implementation of universal molecular screening, and close multidisciplinary collaboration. These issues form the basis for the therapeutic implications discussed in Section 6 and the future perspectives outlined in Section 7.

## 6. The Significance of Hereditary Predispositions for Treatment

The identification of a hereditary predisposition to endometrial cancer influences both the choice of therapeutic strategy for the patient and preventive management in her family. This has implications in several dimensions: oncologic treatment, surgical prophylaxis, monitoring of other organs, and genetic counselling.

### 6.1. Immunotherapy for MSI-H/dMMR—Effectiveness of Immune Checkpoint Inhibitors

Endometrial cancer with microsatellite instability-high status or dMMR represents a distinct group of tumours characterized by a high mutational burden, abundant neoantigen load, and pronounced T-cell infiltration. These biological features favour a strong immune response and underpin the efficacy of ICIs targeting PD-1 and PD-L1 [11]. In this context, immunotherapy has become a key component of treatment for patients with both hereditary predispositions (such as Lynch syndrome) and sporadic MSI-H/dMMR cases [129].

The most robust data originate from the phase II KEYNOTE-158 trial (NCT02628067), which evaluated pembrolizumab in MSI-H tumours, including endometrial cancer [11]. Among patients with advanced, previously treated dMMR/MSI-H endometrial cancer, the overall response rate (ORR) was approximately 48–50%, with durable responses, median progression-free survival (PFS) of 13.1 months and median overall survival (OS) not reached even after over 40 months of follow-up [11,129].

Subsequent trials have confirmed and expanded these findings. The GARNET trial (NCT03981796) investigated dostarlimab, another PD-1 inhibitor, in recurrent or advanced endometrial cancer. In the dMMR/MSI-H cohort, the ORR reached 45.5%, with 85% of responses ongoing at 24 months, highlighting the long-term durability of immunotherapy [42]. Similarly, CheckMate 358 and NCT04165772 evaluated nivolumab, showing an ORR of nearly 59% and a disease control rate exceeding 70%, particularly in tumours with high TMB and robust T-cell infiltration [155]. Preliminary results from the NIVEC study (NCT04734730) demonstrated remarkable neoadjuvant efficacy, clinical complete response in 80% of operable MSI-H/dMMR endometrial cancer after nivolumab monotherapy, suggesting the potential to reduce or even omit surgery in select cases [156].

Combination strategies are also transforming standard treatment. The RUBY trial (NCT03981796), which tested dostarlimab with carboplatin and paclitaxel, demonstrated a significant improvement in PFS and OS, with the greatest benefit observed in the MSI-H/dMMR subgroup, establishing this regimen as a new frontline standard for advanced or recurrent endometrial cancer [42].

Interestingly, responses vary within MSI-H subtypes. Analysis of pembrolizumab-treated patients revealed the highest response rates in Lynch syndrome-associated and Lynch-like tumours, while lower responses were observed in cancers associated with MLH1 promoter methylation, suggesting biological heterogeneity even among MSI-H tumours [156]. Recent long-term follow-up data (KEYNOTE-158 and GARNET) confirm that many responders maintain durable remissions exceeding three years, with manageable toxicity profiles and no new safety signals emerging over time. These outcomes underscore the transformative role of ICIs in the management of dMMR/MSI-H endometrial cancer [(NCT03981796; NCT04165772)].

Importantly, despite biological differences between Lynch syndrome–associated and sporadic MSI-H/dMMR tumours, current clinical data do not consistently demonstrate clinically meaningful differences in response to immune checkpoint inhibitors based on individual MMR gene genotypes (MLH1, MSH2, MSH6, or PMS2). Instead, the mechanism of MMR inactivation and the resulting tumour immune microenvironment appear to be the dominant determinants of therapeutic response [11,129,155,156].

Nevertheless, approximately half of patients fail to achieve a response, emphasizing the need for improved predictive biomarkers. Beyond MSI-H/dMMR, potential markers include tumour mutational burden [157], a T-cell-inflamed tumour microenvironment [158], and specific somatic alterations such as β2-microglobulin loss [41] and JAK1/JAK2 inactivation [159]. PD-L1 expression alone appears to have limited predictive value in endometrial cancer [129].

In summary, ICIs have revolutionized the treatment of advanced and recurrent MSI-H/dMMR endometrial cancer. The integration of immunotherapy, both as monotherapy (pembrolizumab, nivolumab) and in combination with chemotherapy (dostarlimab + carboplatin/paclitaxel), represents a cornerstone of modern gynaecologic oncology. Ongoing trials (e.g., NCT03981796, NCT04165772, NCT04734730) continue to refine patient selection and explore the curative potential of ICIs in earlier disease stages (Table 10).

### 6.2. Surgical Treatment Strategies

Surgical management in Lynch syndrome must balance cancer risk reduction, organ preservation, quality of life, and systemic therapy options. According to the 2025 ESGO–ESTRO–ESP guidelines, surgical and systemic management of endometrial cancer should be guided by molecular classification, with specific recommendations for POLEmut, MMRd, p53abn, and NSMP subgroups [13]. Evidence and guidelines cover prophylactic hysterectomy ± bilateral salpingo-oophorectomy, colorectal and small bowel resections, gastric and thyroid cancer, and metastatic disease surgery, particularly hepatic and pulmonary metastases, with MSI status and immunotherapy increasingly shaping decisions. Key principles include gene-specific risk assessment, shared decision-making, and multidisciplinary care [112].

#### 6.2.1. Gynaecological Surgery

Women with Lynch syndrome have elevated endometrial and ovarian cancer risks, highest for MLH1/MSH2, intermediate for MSH6, and lowest for PMS2. Prophylactic total abdominal hysterectomy with a bilateral salpingo-oophorectomy after completion of childbearing (commonly between 35 and 45 years of age) reduces risk and is guideline-supported. Timing should reflect gene, family history, and reproductive choices. Surgery removes the need for imperfect surveillance but requires consideration of surgical menopause and hormone replacement [160].

#### 6.2.2. Colorectal and Small Bowel Surgery

Lynch syndrome carriers are prone to synchronous and metachronous colorectal cancer. Extended colectomy reduces metachronous risk, especially in younger MLH1/MSH2 carriers, but compromises bowel function. Segmental colectomy with intensive surveillance is appropriate for older patients, rectal primaries, or those prioritising quality of life. Small-bowel adenocarcinoma risk is increased, but absolute incidence remains low; prophylactic resection is not indicated. Surgery follows standard oncological principles [161,162,163].

**Table 10:** Drugs and Their Effectiveness in Clinical Trials (MSI-H/dMMR Endometrial Cancer).

Study/Drug	Population	ORR (Overall Response Rate)	PFS (Progression-Free Survival)	OS (Overall Survival)	Durability/Notes	Based on
KEYNOTE-158/Pembrolizumab	Advanced or recurrent endometrial cancer; MSI-H/dMMR; post-chemotherapy	≈48–50%	Median 13.1 months	Median not reached (>40 months’ follow-up)	Many responses ongoing ≥ 36 months	[11,129,164]
Phase II/Nivolumab	Endometrial or ovarian cancer; dMMR/MSI-H	58.8%	PFS24 = 64.7%	Not reached (follow-up ongoing)	Disease control rate > 70%	[155,165]
NIVEC (Neoadjuvant Nivolumab)	Resectable MSI-H/dMMR endometrial cancer; pre-surgery	Clinical CR 80%	-	-	Early neoadjuvant data; organ-sparing potential in select cases	[156,166]
GARNET/Dostarlimab (monotherapy)	Recurrent/advanced endometrial cancer; dMMR/MSI-H	≈45.5%	NR (not primary endpoint)	Not mature	≈85% of responses ongoing at 24 months; median DOR not reached	[42,167]
RUBY/Dostarlimab + Carboplatin-Paclitaxel vs. Chemo	Advanced or recurrent endometrial cancer; MSI-H/dMMR subgroup	>70% (combination arm; study not powered for ORR)	Significant benefit vs. chemo alone (HR for PFS markedly <1)	OS benefit emerging; data maturing	Largest benefit seen in MSI-H/dMMR subgroup	[116,167]
MSI-H Subtype Subanalysis/Pembrolizumab	MSI-H endometrial cancer: MLH1-methylated vs. Lynch-like vs. Lynch syndrome	MLH1-methylated ≈75%; LS/LLS up to ≈100% (small cohorts)	-	-	Heterogeneity within MSI-H; LS/LLS show highest ORR	[168]

#### 6.2.3. Gastric Cancer

Risk is higher in MLH1/MSH2 carriers and in high-incidence regions, but prophylactic gastrectomy is not recommended. Instead, targeted surveillance with oesophagogastroduodenoscopy, biopsies, and *H. pylori* testing/treatment is suggested in selected carriers. Established cancers are managed per standard oncological resection and lymphadenectomy [169].

#### 6.2.4. Thyroid Cancer

Thyroid neoplasia is not Lynch syndrome-defining. Evidence is limited to case reports and small series, and routine surveillance or prophylactic surgery is not supported. Thyroid cancers should be managed according to endocrine oncology standards, while alternative familial syndromes should be considered if history suggests [170].

#### 6.2.5. Metastatic Disease

Surgical resection of hepatic or pulmonary metastases remains potentially curative in selected colorectal cancer patients, depending on resectability, patient fitness, tumour biology, and systemic options. Hepatic resections (with ablation, staged resections, or perioperative chemotherapy) and pulmonary metastasectomy can yield long-term survival. MSI status influences systemic therapy but not surgical principles [171]. According to immunotherapy paradigms, dMMR/MSI-H metastatic cancers respond well to ICIs, altering surgical planning. ICIs can convert unresectable disease to resectable, support neoadjuvant approaches, and demand careful coordination due to atypical responses and toxicity [172].

In summary, surgery in Lynch syndrome also reflects precision medicine: strategies must align with genotype, cancer type, and stage, patient preferences, and evolving systemic therapies. Unresolved questions include optimal colectomy strategies by gene, appropriate gastric surveillance protocols, and integration of ICIs with metastasectomy. Prospective studies and registries are needed, but current care should follow consensus guidelines, gene-specific risk estimates, and multidisciplinary shared decision-making. The ESGO–ESTRO–ESP 2025 guidelines emphasize this multidisciplinary and gene-adapted approach, positioning molecular profiling as an integral part of surgical and systemic decision-making [13].

### 6.3. Monitoring and Secondary Cancer Screening (Colon, Ovary)

Patients with Lynch syndrome and other hereditary predispositions to endometrial cancer require not only consideration of primary cancer treatment strategies but also a long-term surveillance program for secondary cancers—primarily colorectal cancer and ovarian cancer. These programs are designed to enable early detection of precancerous lesions and early-stage tumours, thereby allowing preventive interventions that significantly reduce morbidity and mortality [12,13]. The NCCN 2024 Guidelines for Genetic/Familial High-Risk Assessment recommend tailored surveillance protocols for individuals with Lynch syndrome, specifying organ-specific intervals and age thresholds for colonoscopy, gynaecologic surveillance, and risk-reducing surgery [6].

#### 6.3.1. Colorectal Cancer

In individuals with Lynch syndrome, the risk of developing colorectal cancer is significantly elevated, and tumours tend to occur at an earlier age and are more often located in the proximal (right) colon. Therefore, colonoscopy remains the primary surveillance method [173]—endoscopic examinations are recommended over sigmoidoscopy, and procedures should be performed under high-quality conditions (HD endoscopes, experienced endoscopists). Data from numerous analyses suggest that regular colonoscopy reduces both the incidence of colorectal cancer and colorectal cancer-related mortality in Lynch syndrome mutation carriers [174].

Regarding the age at initiation and surveillance intervals, traditional guidelines recommended starting at 20–25 years of age and performing colonoscopies every 1–2 years (Table 11) [174]. However, newer data and simulation models indicate the need to consider the genetic MMR subtype: carriers of MLH1/MSH2 mutations usually require earlier and more intensive surveillance (intervals of 1–2 years), whereas for MSH6 and PMS2 carriers, later initiation and longer intervals (e.g., starting at 35–40 years with 2–3 year intervals) may be appropriate, reflecting lower penetrance and later age of onset in these genes (Table 12). Decisions should be personalized, based on family history, individual risk, and test availability [175].

#### 6.3.2. Ovarian Cancer

The risk of ovarian cancer in Lynch syndrome is moderate (lower than in BRCA-associated syndromes), and Lynch syndrome-related tumours are more often diagnosed at early stages compared to BRCA-related cancers. Nevertheless, evidence for the effectiveness of ovarian screening (transvaginal ultrasound [TVUS] and CA-125 testing) in preventing mortality is limited and inconclusive [182]. Systematic reviews and national guidelines indicate that routine screening to reduce mortality is not well supported and is not generally recommended as an effective method for decreasing death rates [183]. In practice, some centres offer targeted surveillance (TVUS + CA-125) from approximately 30–35 years of age for women who have not undergone prophylactic surgery, with the caveat that the effectiveness of this approach is limited [175].

In clinical practice, counselling regarding risk-reducing hysterectomy with bilateral salpingo-oophorectomy (RRH/BSO) after completion of reproductive plans is crucial (Table 10). Risk-reducing surgery significantly lowers the risk of endometrial and ovarian cancer in Lynch syndrome mutation carriers and is often recommended as a prophylactic option, particularly for carriers of MLH1/MSH2 mutations and those who have completed childbearing [184]. The decision to undergo surgery should consider age, reproductive preferences, the impact of surgically induced menopause, and potential benefits and risks (e.g., quality of life implications, need for hormone replacement therapy) [185].

*Practice and recommendations:*

In practice, individualization of surveillance and preventive strategies is recommended. Key elements of this approach include:

Early referral to a genetic counselling clinic after confirmation of a mutation or strong suspicion—this allows discussion of risk, surveillance options, prophylactic surgery, and reproductive planning [186].

Multidisciplinary planning involving a gynaecologic oncologist, gastroenterologist/endoscopist, geneticist, and psychologist—to optimize the schedule of examinations and consider surgical interventions [187].

Education of patients and their families about alarm symptoms (e.g., abnormal uterine bleeding, changes in bowel habits, blood in stool, abdominal pain), the benefits and limitations of screening, and the impact of prophylactic surgery on reproductive health and metabolism [186,188].

Consideration of the genetic variant (MLH1/MSH2 vs. MSH6/PMS2) when planning the age of initiation and interval of examinations, with possible adaptation of recommendations based on the latest models and evidence [175].

### 6.4. Importance for Family Members—Genetic Counselling, Cascade Testing

The diagnosis of Lynch syndrome in a patient with endometrial cancer has important implications not only for the patient herself but also for her relatives. Due to the autosomal dominant inheritance pattern, each first-degree relative has approximately a 50% risk of inheriting a pathogenic mutation in one of the MMR genes. Implementation of genetic counselling and cascade testing allows identification of at-risk individuals and enrolment in an appropriate cancer surveillance program, which can significantly reduce morbidity and mortality in families affected by this syndrome [189]. Recent population-based data confirm that systematic cascade testing within Lynch syndrome families substantially improves early cancer detection and prevention outcomes [126]. This approach is consistent with the recommendations of the NCCN 2024 Guidelines, which underline the importance of cascade testing, early identification of at-risk relatives, and structured genetic counselling pathways [6].

Genetic counselling plays a key role in this process. It includes collecting a detailed family history, constructing a pedigree, and assessing the likelihood of a hereditary cancer predisposition. It is essential to explain the inheritance mechanism, the potential clinical consequences of mutation carriage, and the available prevention and surveillance strategies. Furthermore, qualitative research highlights that effective counselling must also address emotional and social dimensions, including anxiety, family communication, and reproductive decision-making [128]. Counselling should also address psychological and social aspects, such as fear of stigmatization, the impact of test results on family planning, and potential difficulties in communicating information to other family members [127,189].

A key element of the strategy is cascade testing, which involves sequential genetic testing of relatives of an individual identified as a mutation carrier (the proband). This approach allows for the early identification of mutation carriers who require intensified surveillance, while simultaneously reassuring those who did not inherit the mutation. However, studies show that the actual uptake of cascade testing is low—in many families, fewer than half of eligible relatives undergo testing. Barriers include logistical and psychosocial factors, with recent evidence underscoring disparities in participation by gender, age, and socio-economic status [190]. Other obstacles include communication difficulties within families, lack of awareness about risk, limited access to genetic counselling, and emotional or social concerns [127]. The literature emphasizes that the effectiveness of cascade testing depends on integrating genetic activities into routine clinical practice. Automatically referring endometrial cancer patients with dMMR/MSI-H features to genetic consultations increases the detection rate of Lynch syndrome cases and facilitates the initiation of testing for relatives [191]. It is also important to support patients in communicating information to their families, for example, through educational materials, specially prepared brochures, or tele-genetics, which facilitate communication and increase the participation of relatives in testing [192].

## 7. Future Directions and Challenges

### 7.1. Integrating Genetic Testing into Standard Clinical Practice

Modern management of patients with endometrial cancer increasingly acknowledges the role of hereditary cancer predispositions, particularly Lynch syndrome. The integration of genetic testing into routine clinical practice is essential for the early identification of mutation carriers, enabling the implementation of appropriate surveillance and preventive strategies and ultimately improving outcomes not only for patients but also for their families [193].

Current guidelines recommend universal screening of all endometrial cancer cases for mismatch repair deficiency (dMMR) and microsatellite instability (MSI). Initial assessment using immunohistochemistry and molecular testing facilitates the identification of patients who should be referred for further genetic evaluation, including MMR gene sequencing and MLH1 promoter methylation analysis [193]. This approach allows the detection of Lynch syndrome in individuals who do not meet classical clinical criteria, underscoring the growing importance of routine genetic testing in standard oncologic care.

Cost-effectiveness analyses indicate that a universal testing strategy for patients with endometrial cancer is more economical than a selective approach based solely on age or family history. Initial IHC screening combined with MLH1 promoter methylation testing, followed by targeted MMR gene sequencing, enables both accurate identification of mutation carriers and efficient allocation of genetic and preventive resources [194].

Despite these advantages, several challenges hinder the widespread implementation of genetic testing in clinical practice. These include insufficient awareness of cancer genetics among healthcare professionals, limited access to genetic counselling services, and emotional or social barriers affecting patients’ willingness to undergo testing or participate in cascade testing within their families [153].

The literature emphasizes that the effectiveness of cascade testing depends on the close integration of genetic services into routine clinical workflows. Automatic referral of endometrial cancer patients with dMMR/MSI-H tumours to genetic counselling significantly increases the detection rate of Lynch syndrome and facilitates the initiation of testing among at-risk relatives [189].

Supporting patients in communicating genetic risk information to family members is also essential. Tools such as educational materials, dedicated information leaflets, and tele-genetic consultations have been shown to improve communication and increase participation of relatives in genetic testing programs [195].

Overall, effective integration of genetic testing into standard care requires not only access to appropriate diagnostic tools but also systematic education of healthcare professionals, structured genetic counselling pathways, and patient-centered support for family communication. Together, these measures enable early identification of mutation carriers, implementation of evidence-based surveillance programs, and informed decision-making regarding preventive strategies, ultimately translating into improved clinical outcomes [193].

### 7.2. Costs and Accessibility of Testing Within the Healthcare System

Implementation of genetic testing in the diagnosis of endometrial cancer, particularly for Lynch syndrome, involves both economic and organizational considerations. The literature indicates that universal testing of endometrial cancer patients for dMMR and MSI is cost-effective and provides benefits through early identification of germline mutation carriers [196].

The cost of this approach includes initial tests—immunohistochemistry and MLH1 methylation assessment—as well as further MMR gene sequencing in cases of abnormal results. Economic analyses show that the total cost of the universal testing strategy in the UK was approximately £5459 per quality-adjusted life year, which falls within acceptable cost-effectiveness thresholds [196]. Similar conclusions were drawn in the Netherlands, where the Quality-adjusted life year cost for testing patients up to 70 years of age was €5252, also supporting the strategy as cost-effective [197].

The costs of individual diagnostic steps vary. Immunohistochemical testing and MLH1 promoter methylation assessment are relatively inexpensive, whereas MMR gene sequencing and result interpretation generate a higher financial burden [194].

Despite these costs, early identification of mutation carriers allows for more effective implementation of colorectal surveillance, risk-reducing surgery, and appropriate therapy planning, which in the long term reduces expenses associated with treating advanced cancer.

The availability of genetic testing within healthcare systems varies significantly by country and region. In Western Europe, particularly in the UK and the Netherlands, testing for Lynch syndrome is increasingly being incorporated into diagnostic standards for all patients with endometrial cancer [196].

In practice, this means automatic referral for molecular testing and genetic consultations, which increases the detection rate of Lynch syndrome cases and enables the initiation of cascade testing within families.

The literature emphasizes that integrating genetic testing into routine clinical practice, despite cost and organizational challenges, is cost-effective and provides measurable health benefits. It enables early identification of mutation carriers, implementation of preventive measures and cancer surveillance, and improvement of treatment outcomes, while also contributing to a more efficient use of healthcare system resources [196,197].

In Poland, the availability of such testing may be limited, particularly in smaller medical facilities. This, therefore, requires further development of diagnostic infrastructure and education of healthcare personnel in cancer genetics.

In summary, integrating genetic testing into the diagnosis of endometrial cancer is not only cost-effective but also crucial for the early detection of Lynch syndrome and other hereditary predispositions. However, this requires appropriate investment in diagnostic infrastructure and healthcare personnel education to ensure that patients have access to modern and effective diagnostic methods.

### 7.3. Education of Patients and Physicians About Hereditary Cancer Forms

Early recognition of hereditary cancer predispositions, such as Lynch syndrome, largely depends on the awareness of both patients and treating physicians. Education in this area is a key element of effective prevention and diagnosis. Studies indicate that women with endometrial cancer have limited knowledge about the possibility of a hereditary basis for their disease, which significantly reduces their willingness to participate in genetic testing and cascade testing within families [195].

Similarly, the awareness of gynaecologists and oncologists regarding indications for Lynch syndrome testing is often insufficient. Survey analyses among specialists have shown that knowledge of current recommendations for universal testing for dMMR is incomplete, resulting in patients being referred for genetic consultation too infrequently [198]. Gaps in clinician education can lead to diagnostic delays and, consequently, missed cases of Lynch syndrome.

The literature emphasizes that effective patient education should be based on multistep interventions, such as providing understandable informational materials, using brochures and infographics, and leveraging telemedicine and online consultations, which facilitate knowledge transfer and encourage participation in cascade testing [153,195].

At the same time, training for physicians should cover not only genetic knowledge but also practical aspects of result interpretation and strategies for discussing findings with patients and their families [199].

An important component of education is also psychological and communication support. Patients who receive a diagnosis suggesting the possibility of a hereditary predisposition often experience anxiety related to the implications for their family. Therefore, it is recommended that education be closely integrated with genetic counselling and provide tools to facilitate the communication of information to relatives [195].

In summary, increasing awareness among patients and physicians about hereditary forms of endometrial cancer is crucial for improving the effectiveness of diagnosis and prevention. Multichannel education, including direct consultations, multimedia materials, and training for healthcare personnel, helps increase the detection of Lynch syndrome, facilitates the implementation of cascade testing, and contributes to improved treatment outcomes.

### 7.4. Development of “Hereditary Panels” and Multi-Gene Testing

The rapid advancement of next-generation sequencing technology has enabled the implementation of multigene testing, allowing the simultaneous analysis of multiple genes associated with cancer predisposition. In the context of endometrial cancer and Lynch syndrome, this is particularly important because, in addition to the classical MMR genes (*MLH1*, *MSH2*, *MSH6*, *PMS2*, *EPCAM*), mutations are increasingly being identified in other DNA repair genes that may also increase cancer risk [133].

Traditionally, diagnostics focused on sequencing individual genes based on the clinical suspicion of a specific hereditary syndrome. Multigene panels now allow for a comprehensive assessment, increasing diagnostic sensitivity and enabling the identification of so-called “overlapping syndromes”, where mutations in different genes can lead to similar clinical phenotypes [108,132].

This approach allows for the detection of patients who do not meet classical Lynch syndrome criteria but still have an elevated cancer risk.

One of the main advantages of multigene panels is the identification of variants in non-MMR genes, such as *BRCA1/2*, *PTEN*, *POLE*, or *POLD1*, which may also be associated with increased risk of endometrial cancer [2]. Including these genes in testing expands the spectrum of patients eligible for surveillance, but simultaneously introduces challenges related to the interpretation of variants of uncertain significance, requiring specialized bioinformatics and genetic support.

Another aspect of panel development is its growing economic accessibility. The cost of Next-generation sequencing testing has significantly decreased over the past decade, allowing for broader implementation in clinical practice [194]. Nevertheless, in many healthcare systems, access to multigene panels remains limited and dependent on reimbursement policies and laboratory infrastructure.

The literature also emphasizes the importance of multigene testing for family members of patients. The implementation of panels enables cascade testing not only for classical Lynch syndrome mutations but also for other DNA repair genes. This allows for better personalization of surveillance and preventive measures in families at elevated cancer risk [133,200]. Recent molecular studies have also demonstrated that multigene testing contributes to refining molecular classification and prognostic stratification in endometrial cancer, linking certain gene signatures (e.g., POLE, POLD1, PTEN, and MMR variants) to distinct clinical outcomes and therapeutic responses [120].

In summary, the development of hereditary panels represents a significant step toward more comprehensive diagnostics for cancer predisposition. In endometrial cancer and Lynch syndrome, it enables earlier detection of cases that would have been missed using traditional criteria, but it requires parallel advances in variant interpretation, genetic counselling, and systemic support.

### 7.5. Emerging Biomarkers for Screening, Diagnosis, and Prognosis

In addition to established mismatch repair (MMR) immunohistochemistry and microsatellite instability (MSI) testing, several emerging biomarkers are increasingly recognized as promising tools for improving screening, diagnosis, prognostication, and treatment stratification in endometrial cancer.

From a screening and diagnostic perspective, refined MMR testing strategies—including two-antibody immunohistochemical panels (PMS2/MSH6)—have demonstrated high sensitivity while reducing cost and complexity, supporting broader implementation in routine pathology workflows [115,116,117,118]. MLH1 promoter methylation analysis has emerged as a critical adjunct biomarker to distinguish sporadic MSI-H/dMMR tumors from Lynch syndrome–associated cancers and to identify rare cases of constitutional MLH1 epimutation [132,200]. Somatic MMR alterations detected by next-generation sequencing (NGS) further define the subgroup of “Lynch-like” tumors, reducing diagnostic ambiguity in patients lacking germline pathogenic variants [114,121,131,151].

Prognostic biomarkers are increasingly informed by molecular classification. POLE and POLD1 exonuclease-domain mutations identify an ultramutated subgroup associated with excellent prognosis and potential treatment de-escalation [107,109,150,201]. Integration of TCGA-based molecular subtyping has refined risk stratification beyond histopathology alone and is now incorporated into modern FIGO classifications [109,150,202].

Regarding predictive biomarkers, dMMR/MSI-H status remains the strongest predictor of response to immune checkpoint inhibitors (ICIs) [149]. However, emerging data suggest that additional factors—including tumor mutational burden (TMB), immune gene expression signatures, and the mechanism of MMR inactivation (germline vs. somatic vs. epigenetic)—may further modulate immunotherapy responsiveness [107,155,157,158]. Notably, POLE-mutated tumors display high immunogenicity and may represent an expanded population benefiting from immunotherapy despite being microsatellite stable [107].

Finally, future biomarker directions include germline multigene panel testing for comprehensive hereditary cancer risk assessment [108,133,200], advanced NGS-based workflows including whole-genome sequencing [136], and emerging technologies such as liquid biopsy and CRISPR-based diagnostic platforms, which may enable non-invasive disease monitoring and earlier detection in high-risk populations [137].

## 8. Conclusions

Hereditary endometrial cancer represents a relatively small yet clinically meaningful subset of uterine malignancies. It arises predominantly from germline mutations in DNA MMR genes—*MLH1*, *MSH2*, *MSH6*, *PMS2*, and *EPCAM*—resulting in MSI and accumulation of replication errors in key regulatory genes. These molecular alterations drive carcinogenesis in endometrial epithelial cells.

The genetic transmission follows an autosomal dominant model, consistent with Knudson’s two-hit hypothesis, in which the first alteration is germline and the second is somatic. Although Lynch syndrome remains the primary hereditary cause, other rare conditions, such as CS (*PTEN* mutations), polymerase proofreading-associated polyposis (*POLE/POLD1*), Li-Fraumeni syndrome (*TP53*), and occasionally HBOC (*BRCA1/2*), may also predispose to endometrial cancer.

Current clinical practice recommends universal molecular screening of all new endometrial cancer cases using IHC for MMR proteins and MLH1 promoter methylation analysis when MLH1 loss is observed. Next-generation sequencing or MLPA enables definitive germline confirmation and facilitates cascade testing in relatives, allowing for targeted prevention and surveillance.

Importantly, identifying dMMR/MSI-H tumours has therapeutic implications. These tumours show marked sensitivity to immune checkpoint inhibitors (anti-PD-1/PD-L1 therapy), a breakthrough for patients with advanced or recurrent disease. In confirmed Lynch syndrome carriers, prophylactic hysterectomy with bilateral salpingo-oophorectomy after childbearing completion is an effective preventive option.

Despite these advances, persistent barriers include limited access to genetic testing, challenges in variant interpretation (e.g., “Lynch-like” syndromes), and inadequate awareness among clinicians and patients. Effective management of hereditary endometrial cancer thus requires an integrated approach, combining molecular diagnostics, genetic counselling, surveillance, and individualized treatment to improve outcomes and protect future generations through early detection and prevention.

## Figures and Tables

**Table 1:** Estimated cumulative endometrial cancer risk—selected data from clinical/prospective studies (empirical data from Prospective Lynch Syndrome Database and large cohorts).

Gene/Syndrome	Estimated Cumulative Endometrial Cancer Risk by ~70 Years (Examples from PLSD/Cohorts)	Source (Clinical Study)
*MLH1*	~35–40%	PLSD [3]
*MSH2*	~40–50%	PLSD [3]
*MSH6*	~20–44% (increasing with age)	PLSD—particularly high relative endometrial cancer share [3]
*PMS2*	~13–26% (lower penetrance)	PLSD [3]
*PTEN* (Cowden)	~19–28% (in various cohorts)	Clinical cohort studies [41]
*POLE/POLD1* (germline)	Limited data; moderate risk (small cohorts)	Molecular/clinical studies [42,43]

PLSD—Prospective Lynch Syndrome Database.

**Table 2:** Characteristics of MMR protein complexes (based on: Refs. [44,45]).

Complex	Subunit Composition	Main Function	Type of Recognized Errors	Role in DNA Repair
**MutSα**	MSH2 + MSH6	Mismatch detector	Mispaired bases, small insertions/deletions (1–2 nt)	Recognition of mismatches, initiation of the repair process
**MutSβ**	MSH2 + MSH3	Alternative detector	Larger insertions/deletions (up to dozens of nucleotides)	Recognition of complex structural DNA errors
**MutLα**	MLH1 + PMS2	Repair effector	Acts after binding to MutSα/MutSβ	Endonuclease activity, nicking the error-containing strand, coordination of further repair steps
**Entire MMR pathway**	Cooperation of MutSα/MutSβ and MutLα	Removal of DNA fragment containing the error	All types of mismatches detected by MutSα/MutSβ	Recruitment of exonucleases, helicases, DNA polymerase δ, and ligase → synthesis of the correct strand and gap sealing

**Table 3:** Clinical Features of Endometrial Cancer in Lynch Syndrome vs. Sporadic Cases (based on: Refs. [61,62,63]).

Feature	Lynch Syndrome Cancer	Sporadic Endometrial Cancer
Median Age at Diagnosis	~49 years (10–15 years earlier)	~60 years
Age by Gene Mutation	MLH1/MSH2: 39–49; MSH6: 50–60; PMS2: later onset	Not applicable
Tumour Location	LUS: 14–30%	LUS: 3–5%
Histology	Endometrioid: 70–90%–endometrioid: more frequent (serous, clear cell)	Endometrioid: 80–90%-endometrioid: rare
Tumour Grade	Higher frequency of G3	Less frequent G3
BMI	Often lower; obesity less pronounced	Usually higher; obesity is common risk factor
Second Primary tumour Risk	11–33% (colorectal, ovarian, urinary tract)	~5%
Tumour-Infiltrating Lymphocytes	Increased, higher PD-1+/CD8+	Lower, less immunogenic

**Table 4:** The minor and major criteria in the diagnosis of Cowden syndrome [69].

Minor Criteria	Major Criteria
Autism spectrum disorder Colorectal cancer Oesophageal glycogenic acanthosis (≥3) Lipoma (≥3) Intellectual disability (IQ ≤ 75) Renal cell carcinoma Testicular lipomatosis Thyroid cancer (papillary carcinoma or follicular variant of papillary) Thyroid structural lesions (adenoma, adenomatous goiter, etc.) Vascular anomalies (e.g., multiple developmental venous anomalies)	Breast cancer Endometrial cancer Follicular carcinoma of the thyroid gland Gastrointestinal hamartomas (≥3, including ganglioneuromas, excluding hyperplastic polyps) Adult-onset Lhermitte–Duclos disease Macrocephaly (>97th percentile: 58 cm women, 60 cm men) Macular pigmentation of the glans penis Multiple mucocutaneous lesions: • Multiple trichilemmomas (≥3, at least one biopsy-proven) • Acral keratoses (≥3, palmoplantar keratotic pits and/or acral hyperkeratotic papules) • Mucocutaneous neuromas (≥3) • Oral papillomas (≥3, particularly on gingiva and tongue) **Additional requirement**: at least one of macrocephaly, adult-onset Lhermitte–Duclos disease, or gastrointestinal malrotation

**Table 6:** Amsterdam II criteria and Bethesda guidelines revised (based on: Refs. [20,142,143]).

Amsterdam II Criteria	Bethesda Guidelines Revised
Three or more relatives with Lynch syndrome-associated cancer (colorectal, endometrial, small bowel, ureter or renal pelvis): One of the cases must be a first-degree relative of the other two. Two or more successive generations should be affected. One or more must be diagnosed before the age of 50.	colorectal cancer diagnosed in a patient before the age of 50.
	Presence of synchronous or metachronous colorectal cancer or other Lynch syndrome-related tumours, regardless of age.
	Colorectal cancer with microsatellite instability-high histology.
	Colorectal cancer diagnosed in a patient with one or more first-degree relatives with Lynch syndrome-related cancer, with one of the tumours diagnosed before the age of 50.
Familial adenomatous polyposis must be excluded in any colorectal cases.	Colorectal cancer diagnosed in a patient with two or more first- or second-degree relatives with Lynch syndrome-related cancers regardless of age.
All tumours must be verified by pathological examination.	

**Table 7:** Molecular, histologic, and clinical differences between Lynch syndrome-associated and sporadic endometrial cancer (based on Refs. [6,12,13,150]).

Feature	Lynch Syndrome-Associated Endometrial Cancer (Lynch Syndrome-Endometrial Cancer)	Sporadic Endometrial Cancer
Aetiology	Germline mutation in MMR genes (MLH1, MSH2, MSH6, PMS2)	Somatic MLH1 promoter hypermethylation or other mutations
Molecular mechanism	Hereditary MMR deficiency → MSI-H	Epigenetic silencing of MLH1 promoter → MSI-H subset
Most commonly affected genes	MSH2, MSH6 (less often MLH1, PMS2)	MLH1 (promoter hypermethylation)
MSI status	Always MSI-H	MSI-H in ~20–30% (usually MLH1 methylated)
IHC pattern	Loss of one or more MMR proteins (e.g., MSH2/MSH6)	Loss of MLH1/PMS2 due to promoter methylation
Histologic type	Mostly endometrioid (G1-G2), occasionally mixed or serous	Mostly endometrioid (G1-G2)
Tumour location	Lower uterine segment or isthmus	Typically, corpus/fundus
Age at diagnosis	Younger (mean 46–50 years)	Older (mean 60–65 years)
Family history	Positive (colorectal, ovarian, gastric, urinary tract cancers)	Usually negative
Associated cancers	High risk of colorectal, ovarian, gastric, urinary tract cancers	No increased risk of other cancers
Clinical course	May be more aggressive but often detected earlier	Typical course depending on stage and grade
Genetic testing	Indicated (MMR germline mutation testing)	MSI/IHC testing only if MMR deficiency suspected
Therapeutic implications	Responsive to PD-1 inhibitors (pembrolizumab, dostarlimab)	Immunotherapy effective only in MSI-H/MMR-D subset

**Table 8:** Determination of next diagnostic step depending on IHC result.

IHC Result	Interpretation	Next Step
All proteins retained	MMR-proficient → Sporadic endometrial cancer	No further testing unless strong clinical suspicion
Loss of MLH1 + PMS2	Suggests MLH1 promoter hypermethylation	Test for MLH1 promoter methylation
Loss of MSH2 + MSH6	Suggests Lynch syndrome (MSH2 mutation)	Germline testing
Isolated MSH6 loss	Suggests Lynch syndrome (MSH6 mutation)	Germline testing
Isolated PMS2 loss	Suggests Lynch syndrome (PMS2 mutation)	Germline testing

**Table 9:** Interpretation of MSI results.

MSI Result	Interpretation
MSI-H	Typical for Lynch syndrome or sporadic endometrial cancer with MLH1 methylation
MSI-L/MSS (Stable)	MMR-proficient → Sporadic endometrial cancer

**Table 11:** Recommendations for colorectal and ovarian cancer screening (compiled by the authors based on: Refs. [176,177,178]).

Recommendation:
Colonoscopy every 1–2 years, usually starting at 20–25 years of age (Later initiation and longer intervals (e.g., every 2–3 years) may be considered for MSH6/PMS2 carriers with low family penetrance, after individual risk assessment)
Ovarian screening (TVUS + CA-125). Evidence for effectiveness is limited; may be offered as an informational option, but RRH/BSO after completion of reproductive plans remains the only strategy clearly reducing the risk of ovarian and endometrial cancer
Individualization of the plan. Consider gene, age, family history, and patient preferences; management in experienced centres enhances the safety and effectiveness of surveillance.

**Table 12:** Recommendations for secondary cancer screening in patients with Lynch syndrome (based on: Refs. [112,176,179,180,181]).

Cancer	High-Risk Genes (MLH1, MSH2)	Moderate-Risk Genes (MSH6, PMS2)
Colorectal cancer	Colonoscopy from 20 to 25 years of age, every 1–2 years	Colonoscopy from 30 to 35 years of age, every 2–3 years
Ovarian cancer	No evidence for effective screening. TVUS + CA-125 may be considered from 30 to 35 years until RRH/BSO	Similarly, no proven screening efficacy; optional surveillance.

## Data Availability

No new data were created or analyzed in this study. Data sharing is not applicable to this article.

## Abbreviations

The following abbreviations are used in this manuscript:

CNV	Copy number variation
CS	Cowden syndrome
EPCAM	Epithelial cell adhesion molecule
HBOC	Hereditary Breast and Ovarian Cancer syndrome
ICIs	Immune checkpoint inhibitors
IHC	Immunohistochemistry
LFS	Li-Fraumeni syndrome
LUS	Lower uterine segment
MLH1	MutL homologue 1
MLPA	Multiplex ligation-dependent probe amplification
MMR	mismatch repair
MSH2	MutS homologue 2
MSH6	MutS homologue 6
MSI	Microsatellite instability
MSI-H	Microsatellite instability high
MSI-L	Microsatellite instability low
MSS	Microsatellite stable
NCCN	National Comprehensive Cancer Network
ORR	Overall response rate
OS	Overall survival
PFS	Progression-free survival
PHTS	PTEN hamartoma tumour syndrome
PLSD	Prospective Lynch Syndrome Database
PMS2	Postmeiotic segregation increased 2
PTEN	Phosphatase and tensin homolog
RRH/BSO	Risk-reducing hysterectomy with bilateral salpingo-oophorectomy
TMB	Tumour mutational burden
TVUS	Transvaginal ultrasound
VUS	Variants of uncertain significance
